# Confocal Live Imaging of Reproductive Organs Development in *Arabidopsis*

**DOI:** 10.21769/BioProtoc.5177

**Published:** 2025-02-05

**Authors:** Binghan Wang, Amélie Bauer, Andrea Gómez-Felipe, Sylvia R. Silveira, Daniel Kierzkowski

**Affiliations:** Institut de Recherche en Biologie Végétale, Département de Sciences Biologiques, Université de Montréal, 4101 Sherbrooke St E, Montréal, QC, Canada

**Keywords:** Confocal microscopy, Live imaging, Floral organs, Stamen, Gynoecium, Organogenesis

## Abstract

Understanding how multicellular organisms are shaped requires high-resolution, quantitative data to unravel how biological structures grow and develop over time. In recent years, confocal live imaging has become an essential tool providing insights into developmental dynamics at cellular resolution in plant organs such as leaves or meristems. In the context of flowers, growth tracking has primarily been limited to sepals, the outermost floral organs, or the post-fertilization gynoecium, which are easily accessible for microscopy. Here, we describe a detailed pipeline for the preparation, dissection, and confocal imaging of the development of internal reproductive floral organs of *Arabidopsis thaliana* including both the stamen and gynoecium. We also discuss how to acquire high-quality images suitable for efficient 2D and 3D segmentation that allow the quantification of cellular dynamics underlying their development.

Key features

• Fine dissection of tiny and tightly enclosed floral organs.

• Confocal live imaging method allowing long-term observation of plant reproductive morphogenesis.

• Assessing the quality of acquired images for efficient segmentation at cellular resolution in 2D and 3D.

## Background

Flowers are essential for plant reproduction and vital for crop yield. The mature flower of *Arabidopsis thaliana* consists of concentric whorls of floral organs: non-reproductive sepals and petals that surround stamens and carpels (fused together to form gynoecium). The stamen, the male reproductive organ of the flower, consists of anther-producing pollen that is supported by a long filament. The gynoecium, the female reproductive structure, is composed of two fused carpels (forming the ovary) topped by the style and pollen-receiving stigma. After fertilization, the gynoecium develops into fruit, which is crucial for plant propagation.

The development of floral reproductive organs is difficult to observe, as early emerging sepals quickly enclose the initiating stamens and gynoecium making them inaccessible for imaging without dissection. Widely used methods relying on tissue fixing, sectioning, or clonal analysis have provided valuable insights into the development of internal floral organs [e.g., 1–5]. However, these methods lack the spatial and temporal resolution to understand and uncover dynamic developmental processes underlying organogenesis at cellular resolution. While confocal live imaging has been extensively used to follow growth in easily accessible sepals [e.g., 6–10], such an approach has only recently been applied to uncover developmental dynamics in reproductive organs [e.g., 11–13].

In this paper, we describe the confocal live imaging method to observe the growth of *Arabidopsis thaliana* stamen and gynoecium. We detail the procedures for dissecting and exposing internal floral organs and acquiring confocal images of the same sample over several consecutive days. Furthermore, we provide some hints on how to increase the survival rate of the sample and improve the quality of confocal images suitable for 2D or 3D segmentation. Our protocol enables observations with minimal perturbation of the normal in planta morphogenesis allowing long-term quantitative observation of these hidden floral organs at cellular resolution.

## Materials and reagents

**Biological materials**

1. Reproductive organs of 4-week-old *Arabidopsis thaliana* Col-0 carrying a plasma membrane–localized fluorescent marker. Here, we used plants carrying pUBQ10::myr-YFP construct [14]; however, different plasma membrane marker lines can be used

**Reagents**

1. Murashige and Skoog basal salt mixture (Sigma, catalog number: M5524-50L)

2. Sucrose (Fisher, catalog number: S5-3)

3. Murashige and Skoog vitamin solution (Sigma, catalog number: M3900-50ML)

4. Agar (Fisher, catalog number: BP1423-2)

5. Plant preservative mixture (PPM) (Plant Cell Technology, catalog number: 71806-1)

6. 95% denatured alcohol (Fisher, catalog number: HC-1100-1GL)

7. Propidium iodide (PI) (Sigma, catalog number: P4170) (potential carcinogen)

**Solutions**

1. 1/2 MS medium (for 1 L) (see Recipes)

2. 0.1% PPM solution (see Recipes)

3. 70% ethanol solution (see Recipes)

4. 0.1% PI staining solution (see Recipes)

**Recipes**

**1. 1/2 MS medium (for 1 L)**

2.15 g of Murashige and Skoog basal salt mixture

10 g of sucrose

1 mL of Murashige and Skoog vitamin solution

Add deionized H_2_O to 1 L

Adjust pH to 5.8

15 g of agar

**2. 0.1% PPM solution**

1 L of sterile deionized H_2_O

1 mL of plant preservative mixture (PPM)

**3. 70% ethanol solution**

737 mL of 95% denatured alcohol

263 mL of sterile deionized H_2_O

**4. 0.1% PI staining solution (potential carcinogen)**

1 mg of propidium iodide

1 mL of deionized H_2_O

**Laboratory supplies**

1. 35 × 10 mm Petri dishes (SARSTEDT, catalog number: 82.1135.500)

2. Laboratory film (Parafilm^TM^)

3. 200 μL and 1 mL pipette and corresponding tips

4. Precision tweezers with fine point (Dumont No. 5)

5. Syringe needles, 25 G × 1" (BD^®^, catalog number: 305125)

6. Tungsten 1 µm probe tips (Lambda, catalog number: T20-10)

7. Low lint tissue wipe (Kimwipes^TM^, Kimberly-Clark)

8. Deionized H_2_O

9. Scalpel blades (FEATHER, # 15)

10. Surgical tape (Micropore^TM^ tape 3M)

11. Plastic pots, trays, and lids for plant potting (thermoformed square pots with drainage 2.63" × 2.63" × 2.25", no-hole trays 21" × 11" × 2.5", and compatible plastic dome 21.50" × 11" × 2.10")

12. All-purpose growing soil mixture (ASB Greenworld Grower Mix)

## Equipment

1. Autoclave

2. Laminar flow cabinet

3. Dissecting stereomicroscope with a minimum of 4× zoom magnification (Zeiss, model: Stemi 35)

4. Upright confocal microscope (Zeiss, model: LSM800) equipped with long working distance, water-dipping lenses with a good numerical aperture (W Plan-Apochromat 40×/1.0 DIC M27 FWD = 2.5 mm)

5. Growth chamber (Conviron GEN1000)

## Software and datasets

1. Zeiss Zen 2.6 blue edition (Carl Zeiss Microscopy GmbH)

## Procedure

**A. Plant growth**

1. Sow the seeds in the pots with wet soil. Keep a distance of at least 5 cm between seeds. We suggest sowing five seeds per pot, one corner each and one at the center.

2. Accommodate the pots in trays with a 1 cm layer of water and cover with the lids to keep humidity.

3. Keep the trays with lids at 4 °C for 48 h of vernalization.

4. Transfer covered trays to the growth chamber set to long-day conditions (16 h of illumination, around 150 μmol m^-2^·s^-1^), with 60%–70% relative humidity at 22 ± 1 °C.

5. Remove the plastic lids after around 7 days, when the first two leaves are visible to the naked eye.

6. Water trays every other day by pouring around 1 cm of water into the bottom of the tray.

*Note: Plants usually start bolting after around 3 weeks and are ready for dissection after around 4 weeks.*

**B. Preparation of imaging and culture plates**

1. Prepare 1/2 MS medium (see Recipes).

2. Autoclave for 30 min at 120 °C.

3. Let the medium cool to around 50 °C before adding 0.1% PPM solution (see Recipes).

*Note: PPM contains preservative and biocide agents that inhibit the germination of bacteria and fungal spores in in vitro plant cultures.*

4. Under the laminar flow cabinet, fill 1/2–2/3 of the 35 × 10 mm Petri dishes with medium and wait for it to solidify.

5. Wrap the plates with parafilm to avoid contamination and drying.

6. Store plates at 4 °C for up to three months.

**C. Preparation for dissection**

1. Bring the Petri dishes containing the 1/2 MS medium to room temperature to avoid cold shock when placing the dissected sample.

2. Clean working surfaces, tweezers, scalpels, and dissection needles with 70% EtOH solution. It is not necessary to work in a sterile environment like a laminar flow cabinet but ensure your hands, area, and tools are always clean.

3. Place a Kimwipe moistened with deionized H_2_O on the dissection stereoscope stage.

*Notes:*

*a. Other types of tissue wipes may be too soft and may easily tear apart when wet during dissection.*

*b. Be mindful of the amount of deionized H_2_O applied to the tissue. Too much water will soak the sample and make dissection difficult; if not enough water is applied, the sample will dehydrate (Video 1). Re-apply water whenever the Kimwipe is dry.*

**Video 1.**
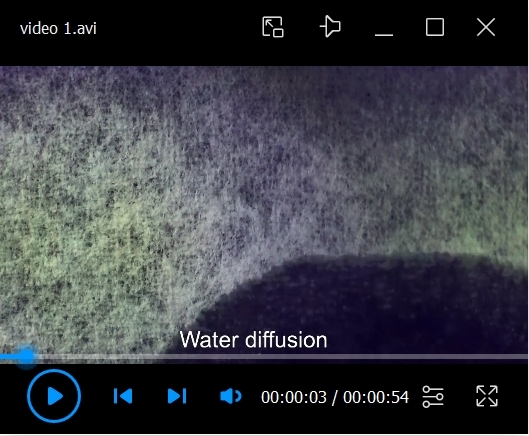
Moistening the tissue wipe. How to prepare for dissection, applying the correct amount of water to the tissue wipe.

**D. Dissection**

*Note: Ensure plants are well-watered, especially one day before the dissection.*

**Figure 1.**
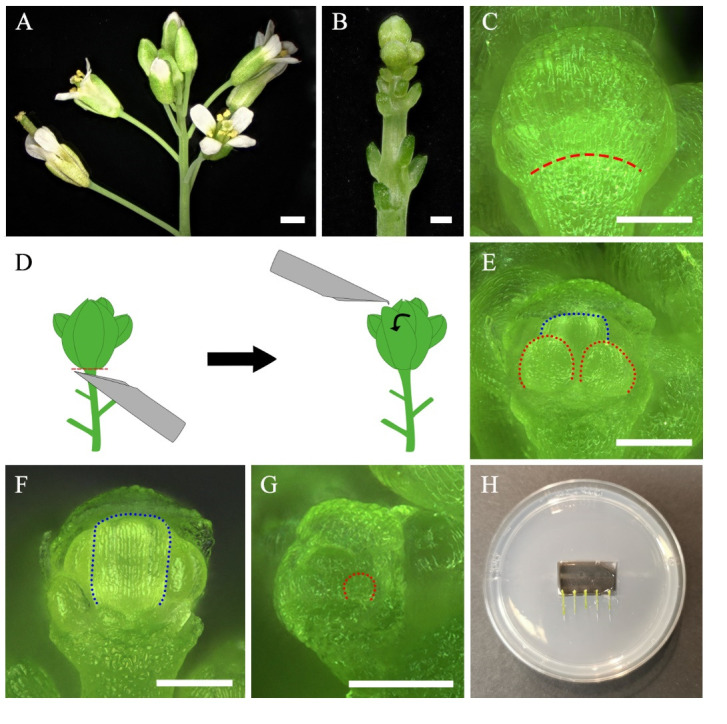
Preparation for live imaging. A–G. Dissection of floral organs. Inflorescence before (A) and after (B) removal of older flower buds. C. Flower bud at floral stage 8. The red dashed line indicates the base of the sepal. D. Schema of flower bud removal using a needle. The red dashed line indicates the location of the cutting. The curved arrow represents the direction of the movement done with the needle to remove the sepal. E. Dissected flower bud after removal of medial and lateral sepals. F. Dissected flower bud after removal of sepals and two long stamens exposing the gynoecium. G. Earlier dissected flower buds after medial sepal removal exposing stamen primordia. Dotted lines indicate the silhouette of developing organs, stamens (red) in (E) and (G), and gynoecium (blue) in (E) and (F). H. Placement of samples in a pre-cut chamber in the in vitro medium. Scale bars = 1 cm in (A), 200 μm in B, 100 μm in (C, E–G).

1. Choose 4-week-old plants (Figure 1A). Pick an inflorescence that has already formed around 10 mature siliques.

*Notes:*

*a. This is a desirable stage because the inflorescence presents enough young flower buds to be selected for dissection while not being surrounded by too many older flower buds. Additionally, at this stage, you have a stem that is long enough to hold and reposition the sample during dissection.*

*b. Older plants will be less vigorous and not suitable for imaging.*

2. Prior to dissection, clean hands with 70% EtOH solution.

3. Cut the inflorescence from the main branch with a clean razor blade or scissors. Make sure you leave around 2–3 cm of the stem so you can hold it with one hand while dissecting with the other.

4. Place the stem on the wet Kimwipe. Grip the stem with the index finger of your non-dominant hand, pressing lightly so as not to crush the sample. Hold the dissection tool (tweezer or needle) with your dominant hand.

*Note: During dissection, you should be able to gently roll the stem by slowly moving your finger left and right without crushing the stem against the tissue (Videos 2 and 3).*

**Video 2.**
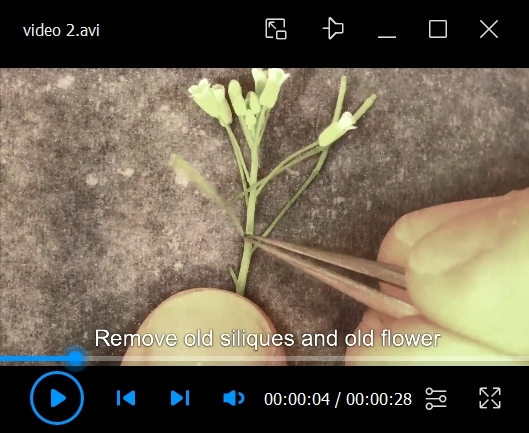
Removal of older flowers. How to remove older flower buds with tweezers while rolling the inflorescence stem.

**Video 3.**
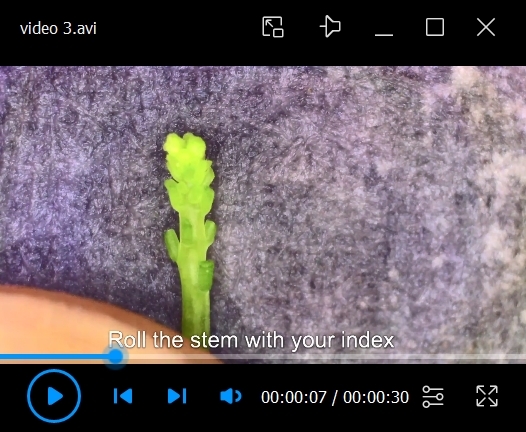
Removal of younger flowers. How to remove younger flower buds with needles while rolling the inflorescence stem.

5. Remove older flowers around the spiral, rolling the inflorescence until you reach the desired stage (Figure 1B and Video 2). The oldest and more distant flowers can be removed by breaking the pedicel with tweezers (Video 2) or cutting with a razor or scalpel blade. Younger flower buds that are closer to the bud of interest should be cut with needles (Video 3).

*Notes:*

*a. The remaining pedicel segments from removed flower buds can later be used to reposition samples avoiding directly touching the stem. However, if the segments are too many and too long, they may hamper the rolling of the stem.*

*b. If you are uncertain what flower bud size corresponds to which floral stage, carefully open the flower buds from outer to inner (bigger to smaller) until you reach the desired stage. A staging reference can be found in Smyth et al. [1], and cellular resolution sizes and landmarks can be found in references [11] and [12].*

6. Roll the stem with your finger to position the flower of the desired stage facing upward.

7. With a dissection needle, scratch or poke the base of the medial sepal as close as possible to the junction between the sepal and pedicel to facilitate sepal detachment (Figure 1C, D and Video 4).

8. Carefully remove the medial sepal without touching other tissues. We suggest using the tip of the needle to lift up the distal part of the sepal and fold it toward the base to break at the base (Figure 1D and Video 4). One or two lateral sepals can be removed in the same way (Figure 1E and Video 4). Alternatively, cut the sepal directly from the base by pushing it up with the tip of the needle (Video 4).

a. If imaging stamens (Figure 1E, F), be careful not to poke the sepal too deep and damage the base of the stamen.

b. If imaging the gynoecium, also remove the two long stamens (Figure 1G and Video 4).

*Notes:*

*a. To facilitate sepal removal, you can gently scratch the tip of the needle onto a hard surface to create a hook. The hook can help pull the tip of the sepals.*

*b. Depending on the floral stage, the two medial petal primordia and lateral stamens may be present but may not yet cover the organs of interest. Keeping them may increase the survival rate of the dissected bud at very early stages. These organs can be removed with tweezers or a needle at any point during the experiment when they extend and partially cover the organ of interest.*

*c. If the organ primordia that you want to remove are too small to be pulled with the tweezers or cut without damaging the tissue behind, or if there is any organ residue attached to the bud, use the dissection needle to poke and damage it. This will impair their growth, preventing them from hiding the organs of interest later in the time-lapse.*

**Video 4.**
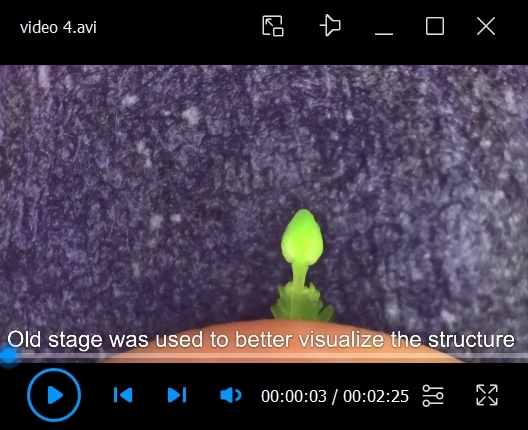
Flower bud dissection. Tricks to remove sepals and stamens that enclose the organ of interest.

9. Gently remove most of the remaining younger flower buds by chopping them off with needles. Only keep a few buds behind the dissected one as physical support (Video 4). This will increase the survival rate of dissected buds. The supporting flower buds can be removed later once the development of the targeted organ stabilizes.

10. Cut the stem around 1 cm from the tip (Video 5).

*Notes:*

*a. To ensure sample health and survival, make sure to have a sharp and clean cut removing the stem extremity that may have been pressed by the finger or hurt by tweezers.*

*b. The stem grows during long-term live imaging. A longer stem brings extra difficulties for positioning. However, cutting the stem afterward may impact the plant’s survival. To avoid having to cut the stem as it grows, try to keep the stem shorter from the start.*

**Video 5.**
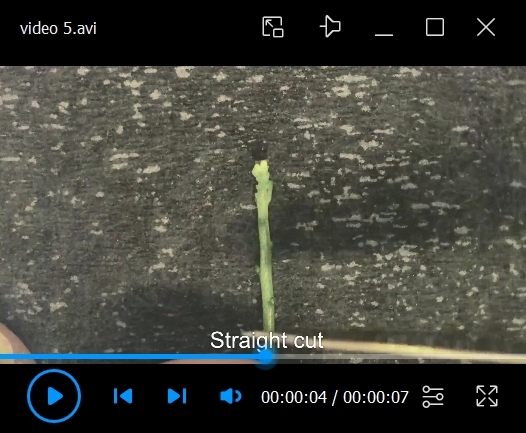
Final chop. How to make the final cut on the stem before mounting the sample.

11. Mount the stem into the MS plate.

a. If imaging the dissected flower bud horizontally, use a scalpel to cut a chamber in the medium and a channel perpendicular to the chamber to place the dissected stem (Video 6). Position the sample perpendicular to the microscope lens (Video 7).

b. If imaging the dissected flower bud vertically, use a needle to make a hole in the agar and insert the sample by the stem. More about choosing sample mounting positions will be discussed in section F, step 4c, note b.

*Notes:*

*a. When mounting and repositioning the sample, touch the pedicel residue and try to avoid touching the stem directly. If touching the stem is necessary, apply as little pressure as possible, rubbing the surface of the stem instead of pinching it.*

*b. Each Petri dish can initially accommodate up to around five inflorescences (Figure 1H). As the samples become big, we suggest reducing the sample number per Petri dish to avoid a longer time of submersion while the other samples on the same plate are being imaged.*

**Video 6.**
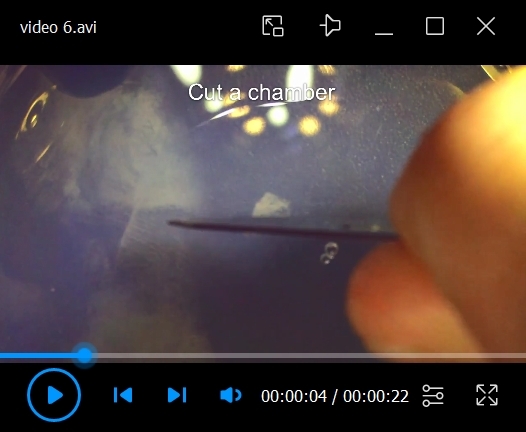
Preparation for sample mounting. How to cut a chamber and a channel in the agar to mount the sample for horizontal imaging.

**Video 7.**
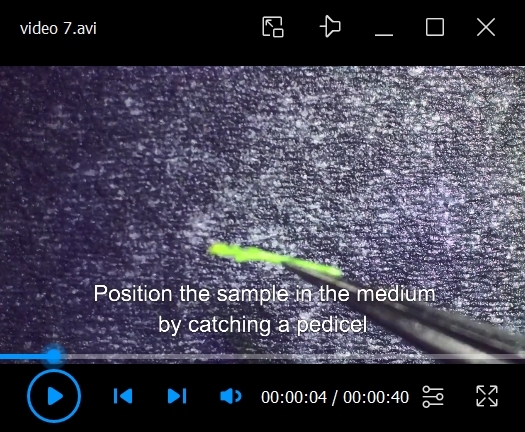
Mounting the sample. How to properly mount the sample on the imaging plate. Tricks to position and reposition the sample by rotating it.

**E. Preparation for imaging**

1. Fill in the cut-out chamber with PPM solution (see Recipes), ensuring all samples are completely submerged in the solution.

*Note: If there is a bubble in front of the organ of interest, use a 200 μL pipette, ensure the tip is targeted on the dissected flower bud, and gently pipette a few times to remove the bubble.*

2. Leave the sample and the medium submerged in the solution for a few minutes (5–10 min) to stabilize in the immersion solution prior to imaging. Plant tissue and agar may expand during water intake, leading to the displacement of the organ during a confocal scan.

**F. Imaging**

**Figure 2.**
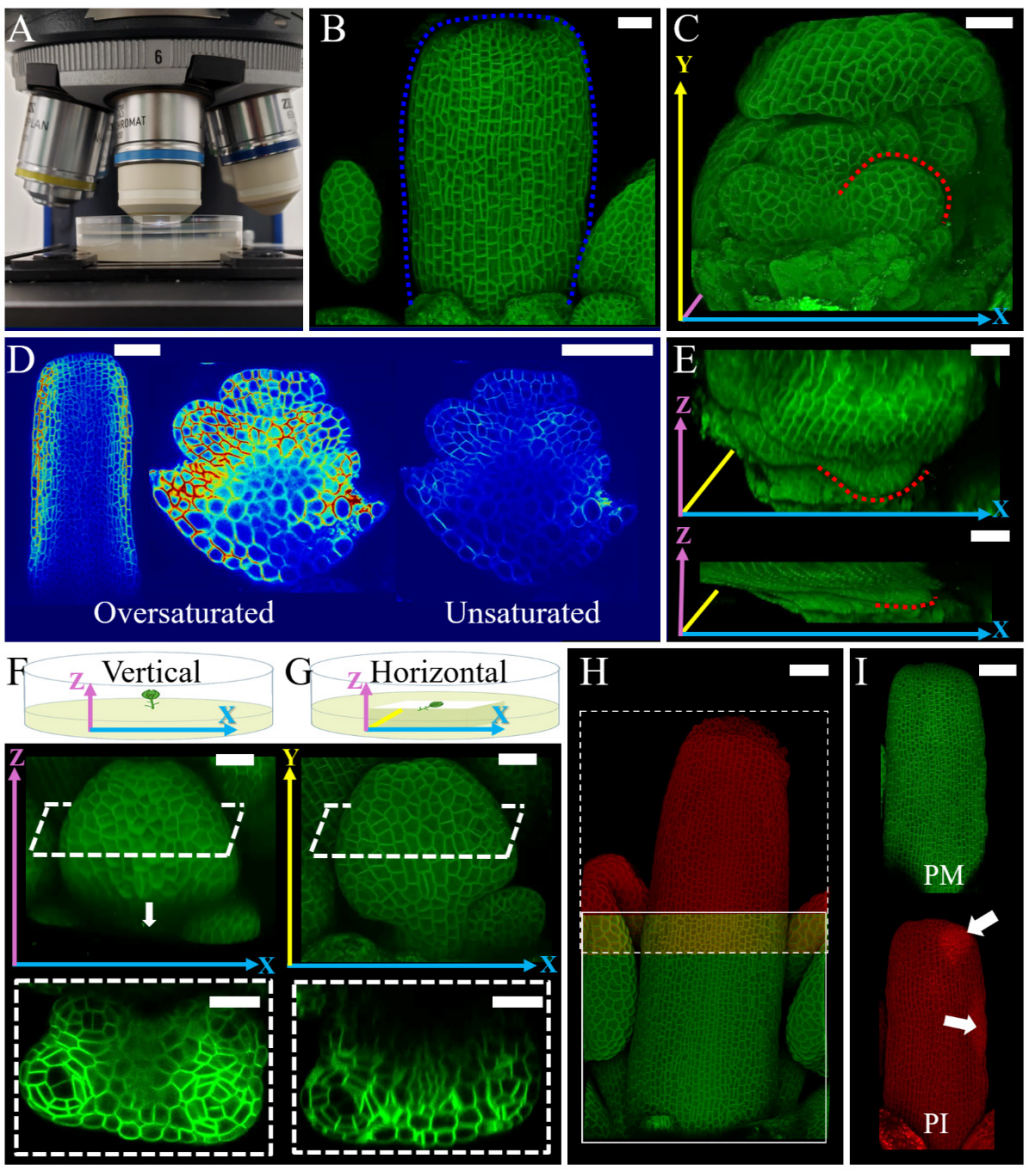
Imaging for growth quantification analysis. A. Setup of a Petri dish with PPM solution under the water immersion objective. B. Confocal image of dissected flower bud exposing gynoecium. Blue dotted lines indicate the same gynoecium indicated in Figure 1F. C. Confocal image of dissected flower bud exposing stamen primordia. Red dotted lines indicate the same stamen primordia indicated in Figure 1G. D. Medial confocal Z-section of dissected flower buds. Gynoecium imaged with oversaturated pixels in the outermost layers and stamen primordia imaged with oversaturated and unsaturated pixels in the inner layers. E. Comparison of Z-axis stack size between non-moving and an extreme case of moving sample. Red dotted lines indicate the same stamen primordia indicated in (C). F–G. Schema of samples mounted vertically (F) and horizontally (G) in the medium and corresponding resulting confocal images. White dashed lines indicate the position of the transversal digital cross-sections of the developing stamen. The arrow in (F) indicates the limitation of signal acquisition in depth. H. Confocal image of a developing gynoecium composed of stitched stacks (green and red). White square outlines the first stack, and the dashed square outlines the second stack. Note the overlapping area. I. Confocal image of stained developing gynoecium with split channels. Plasma membrane marker (PM) (top in green) and propidium iodide (PI) (bottom in red). Arrows indicate damage caused during dissection. Long colored arrows in (C) and (E–G) indicate the axes of the reconstructed confocal images X (blue), Y (yellow), and Z (pink). Scale bars = 20 μm in B, C, and E–G, 50 μm in D, H, and I.

1. Place the Petri dish on the confocal stage and adjust the Petri dish holder (Figure 2A).

2. Select the long working distance water immersion objective (e.g., W Plan-Apochromat 40×/1.0).

*Note: In our system, we use W Plan-Apochromat 40×/1.0, but other water-dipping lenses and magnifications can be used as long as they have a high numerical aperture (we suggest NA > 0.8).*

3. Set up the excitation and signal collection range according to the fluorophore in question (for the plasma membrane marker used here, pUBQ10::myr-YFP excitation was performed using a diode laser with 488 nm and the signal was collected at 500–600 nm).

4. After locating and focusing the sample, switch to live mode and adjust gain and laser power to visualize the contours of cells on the screen. Explore the sample in x, y, and z axes and set up image acquisition parameters as well as the Z-stack range. Acquire your confocal image of the organ of interest at the desired initial developmental stage (Figure 2B, C).

**Critical:** Start your scan from a Z-position above the organ surface (before cells become visible) to make sure the entire organ surface is imaged.

For cell segmentation and growth quantification with the software MorphoGraphX [16] or similar, image acquisition should be done with the following parameters:

a. Acquire 16-bit images. A larger color range helps to obtain more information from darker areas of the image.

b. Define a small Z-step size. For best results, adjust the Z-step size according to the x and y resolution, keeping a voxel size as close as possible to cubic.

*Note: A step size equal to or smaller than 1 μm and 0.5 μm are recommended for 2D and 3D segmentation, respectively.*

c. Optimize image contrast by adjusting laser power and master gain.

For cell segmentation, it is important to have a good contrast between the inside of the cell and cell outlines. In this case, saturating the plasma membrane signal is recommended. Adjust laser power and master gain while monitoring pixel saturation through the range indicator tool from the confocal imaging software (on the Zeiss LSM800 used here, power was around 2% and gain was 650 V). Make sure to make the same adjustments along the Z-stack, either by gradually increasing pixel saturation while scanning or, if your imaging software allows, setting different laser power and master gain values for different Z-positions, prior to starting acquisition.

*Notes:*

*a. For surface (2.5D) segmentation, adjust the laser to oversaturate pixels at the outermost layer and ignore the signal inner layers even if it is weak (Figure 2D gynoecium). However, if inner tissues are also of interest for 3D analysis, adjustments must be made while paying attention to all cell layers (Figure 2D, stamen).*

*b. According to your research goal, you may have to optimize the positioning of the sample for a better signal of the region of interest (Figure 2F, G). Imaging the sample vertically ensures higher signal quality in inner tissues but impairs acquisition of the complete organ length (Figure 2F), while imaging horizontally ensures the acquisition of the entire front organ surface, with lower image quality in internal tissue (Figure 2G).*

**Caution:** Be careful when increasing laser power. Exposure to high laser intensity may cause photobleaching and stresses that are harmful to plant growth.

*c. Keep in mind that if your research goal involves quantifying fluorescence levels and comparing fluorescence between samples, the signal saturation should be avoided.*

*d. There is always a trade-off between image quality for segmentation, laser exposure tolerance, and the available time for the experiment. The experiment setup should be chosen based on the specific goals and work conditions.*

5. After taking the confocal image, click on the 3D display tool from the imaging software to check if the stack acquired represents the realistic 3D shape of the organ.

*Notes:*

*a. The samples may move during image acquisition for several reasons, e.g., strong vibrations, physical movement of the sample, and water absorption by the agar. These movements result in deformed 3D stacks (an example of an extreme case of flattening in the Z-axis is shown in Figure 2E). The data extracted from a deformed confocal stack will not be accurate and representative of reality.*

*b. To avoid movement, we recommend 1) always checking the functioning of the antivibration platform in which the microscope is installed before every imaging session, and 2) respecting the recommended time of incubation prior to imaging (see step 2 section E).*

*c. If strong movement is detected even after the incubation period, transfer the sample to a new Petri dish with a newly cut chamber and channel (see step 11 section D) and carefully position the stem on it.*

6. For samples that are larger than the field of view, acquire multiple overlapping stacks.

*Notes:*

*a. It is recommended that the acquisition be started from one distal corner of the organ. If the complete organ width is acquired in one stack, move the stage longitudinally (along the organ's proximal distal axis) to continue scanning the length of the organ. If the tissue is broader, keep moving and scanning horizontally (medial–lateral axis) until the complete organ width is acquired before moving longitudinally again. Make sure to have a small overlapping area between each stack to guide you in reconstructing the complete image.*

*b. If multiple confocal stacks are acquired for the same sample, use image analysis software of your preference to roughly stitch all the stacks immediately after scanning and confirm the entirety of the sample was imaged. If the final image has any missing pieces, it is still possible to redo the imaging (Figure 2H).*

*c. Depending on scanning speed, Z-stack depth, and Z-step size, each confocal stack may take 2–5 and 5–10 min to scan for 2D and 3D analysis, respectively. If a sample requires overlapping stacks, the acquisition time is multiplied by the total number of stacks (see General notes 5–6).*

**G. Post imaging**

1. Unload the Petri dish from the microscope stage, being careful not to drop the PPM solution on the microscope.)

2. Discard the PPM solution. Remove the excess liquid from the agar chamber with a Kimwipe without touching the analyzed organ.

**Critical:** Keeping even a small volume of liquid around the sample will impair its growth.

3. Verify that the stem is in the correct position (not tilted/incorrect angle). Reposition it if necessary (Video 7).

4. Seal the Petri dish with Micropore tape.

5. Cultivate the dissected samples in an in vitro room/cabinet with the same controlled growth conditions as the growth chamber for the entire duration of the experiment. Place the Petri dish in a position that ensures negative gravitropism (vertical if the sample is horizontal, horizontal if the sample is vertical).

*Note: If the experiment lasts for more than one week, at the end of the first week, place the sample into a new Petri dish with fresh medium to avoid contamination and to provide enough nutrients.*

6. After the defined time interval (e.g., 24 h), restart from step E1.

## Data analysis

**Figure 3.**
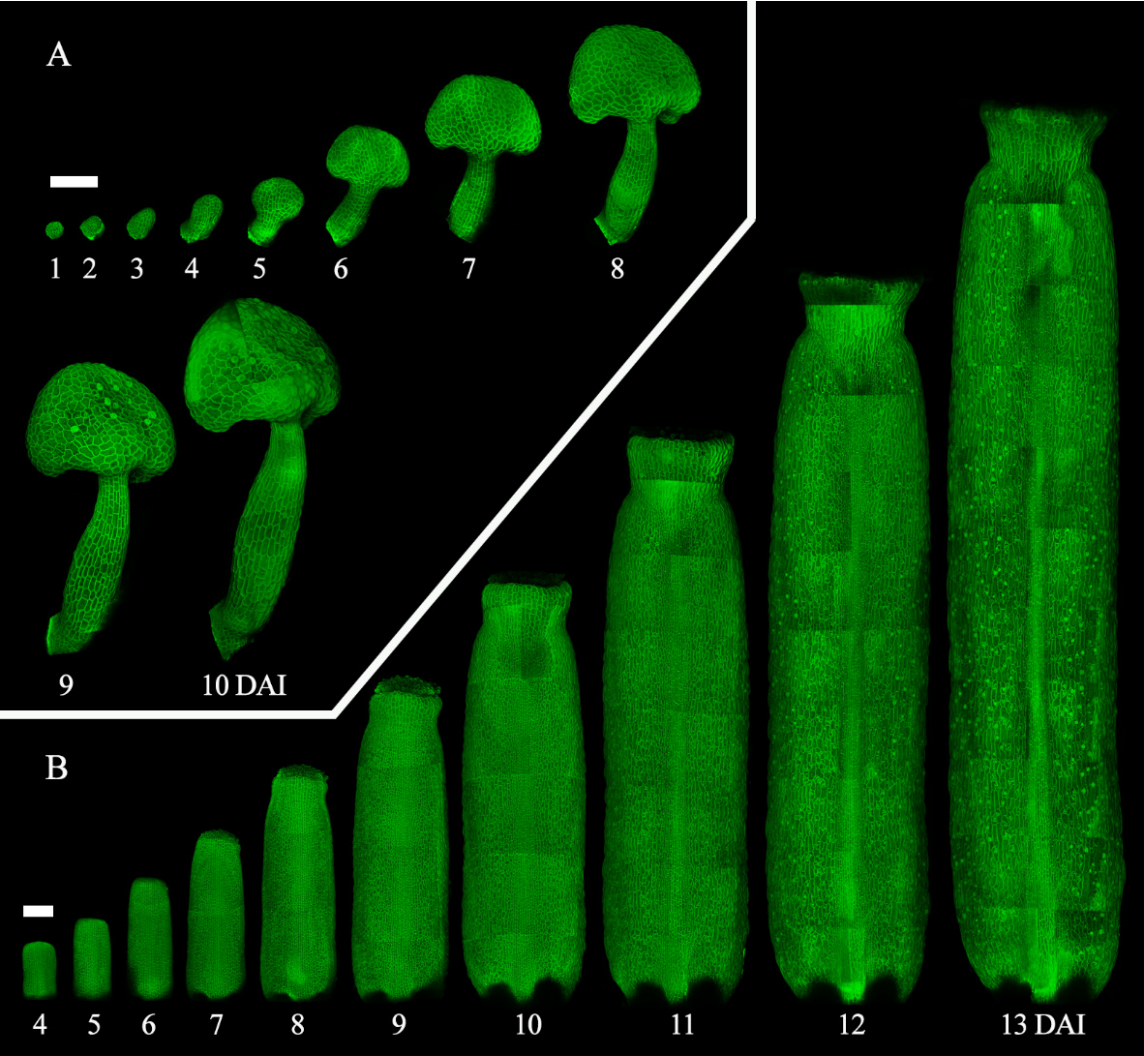
Time-lapse live imaging of *A. thaliana* internal floral organs. A. Confocal images of a time-lapse series of the developing stamen from 1 to 10 days after primordia initiation [11]. B. Confocal images of a time-lapse series of the developing gynoecium from 4 to 13 days after primordia initiation [12]. Scale bars = 100 μm.

In this protocol, we describe in detail a live-imaging method that enables tracking the development of *Arabidopsis* reproductive organs. Unlike previous live-imaging studies, which are generally limited to easily accessible floral organs namely sepals, our protocol allows the detailed imaging of the innermost reproductive floral organs for extended periods of time (up to two weeks). By carefully dissecting the external floral whorls, we expose the internal floral organs and observe their development from early initiation until the establishment of their final morphology, while preserving in vivo–like characteristics of the organogenesis (Figure 3).

Data obtained with this protocol can be used for both 2D [11,12] and 3D [13] cell segmentations and growth quantifications with advanced image analysis software such as MorphoGraphX [15,16]. Furthermore, this protocol may be reproduced to track the development in both wild-type and mutant specimens or samples upon chemical treatments, facilitating the understanding of how molecular factors modify key cellular behaviors during organogenesis. The method was successfully applied using *Arabidopsis* mutants with defects in critical reproductive processes, such as carpel fusion, style identity [12], and sporogenesis [13]. All these mutants survived the conditions imposed by this method, as did the wild type, while retaining their characteristic defective phenotypes. Beyond *Arabidopsis*, we believe that this protocol could be adapted for long-term, quantitative studies of reproductive organ development in other species, which are currently limited to either early primordia initiation [21] or gynoecium shape transformations post-fertilization [22]. Since the method depends on successful in vitro culture, optimization of the culture medium may be necessary, depending on the species' sugar and nutrient requirements. An additional challenge may arise with bigger organ sizes, as larger species may not fit within our recommended setup and will likely extend the duration of the experiments. Overall, this protocol offers a robust and adaptable approach, with the potential to significantly enhance our understanding of morphogenesis in the reproductive organ of the flower.

## Validation of protocol

This protocol or parts of it has been used and validated in the following research articles:

• Silveira et al. [11]. Live-imaging provides an atlas of cellular growth dynamics in the stamen. *Plant Physiology*.

• Gómez-Felipe et al. [12] Two orthogonal differentiation gradients locally coordinate fruit morphogenesis. *Nature Communications*.

• Kierzkowski et al. [13] Mechanical interactions between tissue layers underlie plant morphogenesis. Preprint available at *Research Square*.

## General notes and troubleshooting

1. During several steps of dissection, while manipulating the sample with tweezers and needles, non-intentional damage to the plant tissue may occur. Samples may recover, but such wounds may impair your experiment (e.g., locally affect growth). Therefore, to increase the dissection success rate, it is essential to practice before directly investing time into the time-lapse live imaging. It usually takes one to several weeks before you are able to confidently dissect depending on the initial size of your samples.

2. During the practice period, propidium iodide (PI) staining is a useful tool to check for mistakes in dissection. Because PI penetrates wounded cells, it facilitates spotting the location of tissue damage, which is not always visible with the plasma membrane channel (Figure 2I). If choosing to use this strategy, we recommend staining samples for 5 min with 0.1% PI solution (see Recipes). Staining can be performed before mounting the sample in the medium by applying a few drops of PI solution directly to the dissected flower on a glass slide. Alternatively, after mounting the sample, fill the Petri dish or agar chamber with the 0.1% PI solution. In both cases, after 5 min, rinse it twice with PPM solution before filling the Petri dish for imaging. It is recommended that the acquisition be started from one distal corner of the organ. If the complete organ width is acquired in one stack, move the stage longitudinally (along the organ's proximal–distal axis) to continue scanning the length of the organ. If the tissue is broader, keep moving and scanning horizontally (medial–lateral axis) until the complete organ width is acquired before moving longitudinally again. Make sure to have a small overlapping area between each stack to guide you in reconstructing the complete image.

**Caution:** Propidium iodide is a potential carcinogen and should be handled with care. Avoid contact with skin and eyes by wearing suitable protective clothing, gloves, and eye/face protection. Dispose of the dye safely following local regulations.

3. In some cases, the dissected sample may exhibit limited growth between the first two time points. However, it may recover and develop normally later on. Therefore, it is recommended not to discard the sample immediately but to continue imaging and allow additional time for recovery. This approach can improve the sample survival rate and reduce the overall difficulty of experiments.

4. While this method has been shown to support all developmental stages (Figure 3), it is not entirely non-invasive. Stress from dissection, in vitro cultivation, and laser exposure can impact development. For example, exposing a primordium earlier may lead to earlier cessation of growth. To ensure a comprehensive and accurate time-lapse series, we recommend having as many replicates as possible and then selecting a minimum of three, based on the quality of the series, for the following analysis. When evaluating the quality of a time-lapse series, consider the following criteria: 1) Series that best represent the general developmental patterns expected based on literature and observed frequently across all replicates. 2) Series that capture the full or most of the developmental stages of interest within a single time-lapse. 3) Series with minimal to no cell damage at individual time points. If there are less than three, perform the experiment again following the same principle.

5. If fewer than three replicates are obtained, repeat the experiment to collect more. If needed, combining two independent partially overlapping time-lapse series can serve as a single replicate, offering a comprehensive view of organ development.

6. As samples grow bigger, the time to image one sample increases as days go by. Consider this when scheduling microscope slots and planning how many replicates will be followed at a time. For instance, the gynoecium at 13 DAI shown in Figure 3 is composed of approximately 25 confocal stacks. Therefore, imaging the whole organ at later developmental stages could take more than 1 h for a single sample.

7. Experimental setup decisions should be made by carefully weighing all relevant trade-offs, taking into account the specific conditions of your study and the requirements of your research questions. For instance, consider whether the research group has unrestricted or flexible access to a confocal microscope or if it is essential to capture the entire organ or the full developmental process.

8. The images obtained using this method were shown to be suitable for quantitative analysis in both 2D and 3D using the image analysis software MorphoGraphX [15–16]. The studies that validate this protocol outline some processes and parameters used for image processing, surface extraction, cell segmentation, and growth quantification in their method sections [11–13]. Detailed step-by-step instructions for analysis in MorphoGraphX can be found in several method and protocol publications [17–20]. Additional recommendations, information on the required computer hardware, tutorial videos, and troubleshooting resources are available at https://morphographx.org/. We also suggest visiting https://forum.image.sc/tags/MorphoGraphX and engaging in discussions.
